# The Behavior of Primary Healthcare Doctors Toward Antibiotic Prescriptions for Upper Respiratory Tract Infections

**DOI:** 10.7759/cureus.53298

**Published:** 2024-01-31

**Authors:** Ahlam S Alharbi, Mayar S Alharbi, Kholoud B Almutairi, Raghad M Alsaady, Rouz M Alsaedi, Renad S Alhejaili

**Affiliations:** 1 Family and Community Medicine, Ministry of Health (MOH), Riyadh, SAU; 2 Medicine and Surgery, Al-Rayan Colleges, Al Medinah, SAU

**Keywords:** upper respiratory tract infections (urti), primary health care, ksa:kingdom of saudi arabia, physicians, behavior, antibiotics

## Abstract

Background: Unnecessary prescription of antibiotics for patients with upper respiratory tract infections (URTIs) carries the potential risk to the development of bacterial resistance.

Objective: This study aimed to investigate the behavior of primary healthcare (PHC) physicians toward an antibiotic prescription for URTI, Al-Madinah City, Saudi Arabia in 2021.

Methods: A cross-sectional study was conducted at PHC centers in Al-Madinah City, Saudi Arabia. The study invited all physicians in the randomly selected 28 PHC centers to participate in the study. A master sheet adopted from a researcher done in the Asir region of Saudi Arabia about the pattern of prescription for URTI was used and included data about socio-demographic characteristics and data about presenting symptoms and signs of URTIs, the clinical diagnosis, type of medication prescribed, and duration of treatment also, included data about the factors that press physicians to prescribe antibiotics and their response. The questionnaire was filled out and returned back by 140 physicians. The collected data were analyzed and tabulated using appropriate statistical tests.

Results: The mean age of the studied physicians was 34.4 ± 7.6 years (25-59 years). General practitioners and specialists were 66.4% and 33.6%, respectively. The prevalence of antibiotic prescriptions was 44.3%. The most prescribed antibiotics were amoxicillin (58.6%) and Augmentin (28.6%). Congested tonsils (87.1%), ear discharge (84.2%), and cervical lymphadenopathy (89.3%) were the most clinical factors that affected physicians' decisions to prescribe antibiotics for URTI. The non-clinical factors affecting physicians' decisions include patient request (52.8%) and press (28.5%), with no statistically significant difference detected between general practitioners and specialists.

Conclusion: The study findings indicate the need to develop intervention programs targeting physicians as well as the general population to decrease inappropriate antibiotic prescriptions in primary care centers.

## Introduction

One of the most frequent causes of primary care physician visits is respiratory tract infections (RTIs) [1]. According to a recent study, Saudi Arabia has a 15% prevalence rate of the disease among children [2]. The highest burden is noted in low social demographic regions such as South Asia and sub-Saharan Africa [3]. Other studies indicate that less than 10% of instances are caused by bacteria; most cases have viral causes [4]. The misuse of antibacterial drugs, including their application for treating viral infections such as the flu or common cold, may contribute to the emergence of drug-resistant bacteria. Antibiotic resistance arises when bacteria undergo evolutionary changes that render them resistant to medications designed to eliminate them, thereby complicating the treatment of infections [4]. A recent cross-sectional study conducted in 2015 in the adult and pediatric emergency departments of King Abdullah International Medical Research Centre revealed that the prevalence of antibiotic misuse was 38.7% in adults and 57.8% in children [5].

A study conducted in Jeddah that sought to find the pharmacists' attitudes and practices regarding the dispensing of non-over-the-counter drugs revealed that 97.9% of pharmacists either recommended the drugs or adhered to the patient's preferences. Saudi Arabia has never implemented a policy prohibiting the distribution of these medications without a prescription [6]. A cross-sectional analysis of 327 pharmacies across Riyadh's diverse regions revealed widespread non-prescription antibiotic availability, independent of physician consultations or established medical diagnoses [7]. Another study carried out to ascertain the frequency of non-prescription antibiotic usage comprising 313 participants and revealed that 41.7% of the respondents preferred non-prescription antibiotics while the rest preferred using antibiotics with prescription [8].

Another study that looked at the frequency of antibiotic abuse and the number of nosocomial infections over a six-month period in community healthcare facilities endowed with 174 in Saudi Arabia came to the conclusion that an elevated prevalence of nosocomial infections combined with a widespread antimicrobial misuse should be of great concern to the public health officials in the area [9]. Nonetheless, over 50% of cases of infections of the upper respiratory tract (URTIs) caused by viruses result in prescriptions for antibiotics from doctors [10]. General practitioners initiated the practice of prescribing antibiotics, aiming to reduce illness severity, shorten its duration, and prevent complications such as pneumonia or acute otitis media [11]. There is no solid proof from clinical trials to support this [12,13]. According to previous research, the majority of URTIs are actually self-limiting illnesses best managed with symptomatic therapy; additionally, patients are more likely to experience side effects from antibiotic treatment than to benefit from it [13]. Antibiotic resistance poses a significant threat to human health as it can lead to the failure of standard treatment protocols for bacterial infections [14].

Antimicrobial resistance fosters the emergence of resistant infections and escalates morbidity and mortality. Additionally, it not only poses a threat to individuals but also heightens the likelihood of communities being susceptible to resistant infections [15]. Additionally, according to the study by Mainous et al., treating viral URTIs with antibiotics inappropriately is one of the main factors contributing to the rise of antibiotic resistance [15]. Therefore, this study aims to investigate the behavior of primary healthcare (PHC) doctors in Saudi Arabia concerning antibiotic prescriptions for URTIs, given the high prevalence of URTIs and the substantial misuse of antibiotics in the country.

## Materials and methods

Study design

The research adopted a cross-sectional descriptive study design. It was conducted within primary healthcare centers (PHCC) in Al-Madinah city, Saudi Arabia spanning from March 2023 to September 2023. The study encompassed PHC physicians practicing in Al-Madinah city.

Sampling procedures

This study employed a multistage, cluster random sampling method, selecting seven PHCCs randomly. Consequently, a total of 28 PHCCs were included in the final sample. All physicians working in the PHCC were subsequently invited to participate in the study.

Inclusion criteria

The study invited all physicians Saudi and non-Saudi but excluded pharmacists, technicians, nurses, other staff, and individuals who declined to participate due to ethical considerations.

Study tool

A master sheet was adopted from research done in the Asir region of Saudi Arabia about (the pattern of prescriptions for URTI) with some modifications done to suit the current study; the questionnaire was divided into three sections [16]. The first part has socio-demographic information while the second part includes data about the presenting symptoms and signs of URTIs, the clinical diagnosis, the type of medication prescribed, and the duration of treatment. The third section included data about the factors that press physicians to prescribe antibiotics and their response.

Data collection technique

Before collecting data, a pilot study was conducted involving five physicians, who were subsequently excluded from the main study. The questionnaires were distributed to all physicians working in PHCCs selected in the study in Al-Madinah city. Instructions had been given that the treating physician should enter any patient presenting with a symptom of acute URTIs in the provided questionnaire. Every physician was allotted one-week time to fill up the questionnaire. The questionnaires were collected from 140 physicians representing a response rate of 94.6% (140/148) among the invited physicians in the selected 28 PHCCs.

Statistical analysis

The data gathered from the 140 willing physicians was analyzed using the Statistical Analysis System (SAS) (SAS Institute Inc., Cary, NC) software. Physicians' characteristics were summarized: categorical variables were presented by frequency numbers and percentages, while continuous variables were represented by their mean value alongside the standard deviation (mean ± SD). Also, the most common antibiotics prescribed to URTI patients by PHC were tabulated in frequency number and percentage. Comparison of prescribing antibiotics by its duration, and physicians' characteristics were done using appropriate statistical tests, independent t-test for parametric data, and Chi-square and Fischer exact tests for non-parametric data. P-value ≤ 0.05 was considered as an indicator of statistically significant difference.

Ethical considerations

Prior to commencing the study, formalities were diligently addressed, beginning with securing written permission from the General Supervisor of the joint program of Family Medicine in Al-Madinah. Additionally, official approval letters were acquired from higher authorities within the Ministry of Health. To safeguard participant confidentiality, stringent measures were implemented, ensuring the absolute privacy of all study-related data. Furthermore, the study received the necessary ethical clearance from the Ethical Committee in Al-Madinah, underscoring the commitment to conducting the research with adherence to ethical standards and guidelines.

## Results

Data from a total of 140 PHC physicians were analyzed. Table 1 presents the characteristics of the participant physicians. Their mean age was 34.4 ± 7.6 years (25-59 years). General practitioners and specialists were 93 (66.4%) and 47 (33.6%), respectively. The occupational experience years were less than five years in 43 (30.7%) of the studied physicians and those with more than 10 years of experience were 57 (40.7%). A total of 67 (48%) of the studied physicians were Saudi and those reported following continued medical education in terms of respiratory infection courses were 52 (37.1%).

**Table 1:** Characteristics of the studied physician Data are presented by the mean ± SD or by n (%).

Characteristics*	N(%)
Age in years;	
mean ± SD (Range)	34.4 ± 7.6 (25-59)
Occupational title	
General practitioner	93 (66.4)
Specialists**	47 (33.6)
Occupational years	
< 5	43 (30.7)
5-10	40 (28.6)
> 10	57 (40.7)
Nationality	
Saudi	67 (47.9)
Not Saudi	73 (52.1)
Following continued medical education	
Yes	52 (37.1)
No	88 (62.9)

Table 2 shows the frequency of total patients with URTIs seen by physicians per day. A total of 40 (28.6%) studied physicians reported seeing 1-10 patients per day, 78 (55.7%) reported seeing from 10 to 15 patients per day, and 22 (15.7%) reported seeing more than 15 patients with URTIs per day.

**Table 2:** Total patients with URTIs seen by physicians per day (N= 140) Data has been presented as frequencies(n) and percentages (%). URTIs: upper respiratory tract infections

Patients with URTIs*	N (%)
1-10 patients	40 (28.6)
10-15 patients	78 (55.7)
> 15 patients	22 (15.7)

Table 3 shows the most common antibiotics prescribed to URTI patients by PHC physicians. Amoxicillin was the most common antibiotic prescribed for patients with URTIs as it was reported by 82 (58.6%) of the studied physicians followed by Augmentin as it was prescribed by 40 (28.6%) of the studied physicians. Erythromycin, quinolone, and cephalosporins were not in common use by the studied physicians as erythromycin was prescribed by 10 (7.1%) and quinolone and cephalosporins by 8 (5.7%). No one of the studied physicians reported a prescription of penicillin V.

**Table 3:** The most common antibiotics prescribed to URTI patients by PHC physicians (n=140). Data has been presented as frequencies (n) and percentages (%). URTIs: upper respiratory tract infections, PHC: primary healthcare

Antibiotics*	N (%)
Amoxicillin	82 (58.6)
Augmentin	40 (28.6)
Erythromycin	10 (7.1)
Penicillin-V	0 (0.0)
Quinolines and Cephalosporins	8 (5.7)

Table 4 shows the antibiotic prescription patterns based on professional designations. Among the 93 general practitioners, amoxicillin was the most prescribed antibiotic, prescribed by 60 (64.5%) practitioners followed by Augmentin with 20 (21.5%). Conversely, the specialists exhibited a different trend with 22 (46.8%) prescribing amoxicillin and 20 (42.6%) prescribing Augmentin. Erythromycin showed minimal usage across both groups, 8.6% (n=8) among general practitioners and merely 4.3% (n=5) among specialists. Statistical analysis demonstrated significant discrepancies in antibiotic prescription patterns between the two groups for amoxicillin (p=0.01*) and Augmentin (p=0.04*), emphasizing distinctive preferences in antibiotic choices based on professional roles among PHC physicians.

**Table 4:** The association between PHC physicians and antibiotic prescription behavior (n=140). Data has been presented as frequencies(n) and percentages (%). The P-value is considered to be statistically significant at p-value< 0.05. PHC: primary healthcare

PHC Physicians		Antibiotics Prescribed			
	Amoxicillin	Augmentin	Erythromycin	Quinolones and cephalosporins	P-value
General practitioners (n= 93)	60 (64.5)	20 (21.5)	8 (8.6)	5 (5.4)	0.01*
Specialists N= (47)	22 (46.8)	20 (42.6)	2 (4.3)	3 (6.3)	0.04*

Table 5 shows the most common antibiotics prescribed to URTI patients by PHC physicians by their occupational experience (n=140). There have been statistically significant differences in the commonest antibiotics prescribed by physicians according to their experience years. Amoxicillin and Augmentin were the most prescribed antibiotics among physicians with > 10 years of experience. Although no statistically significant difference, quinolone and cephalosporins were also prescribed more by physicians with > 10 experience years. Erythromycin was significantly prescribed by physicians with 5-10 years of experience.

**Table 5:** The most common antibiotics prescribed to URTI patients by PHC physicians by their occupational experience (n=140). Data has been presented as frequencies(n) and percentages (%). * P-value considered statistically significant at p-value< 0.05. URTI: upper respiratory tract infection, PHC: primary healthcare

Antibiotics	Amoxicillin	Augmentin	Erythromycin	Others
< 5 years	30 (36.5)	9 (22.5)	2 (20.0)	2 (25.0)
5-10 years	19 (23.2)	14 (35.0)	5 (50.0)	2 (25.0)
> 10 years	33 (40.3)	17 (42.5)	3 (30.0)	4 (50.0)
P-value	0.03*	0.04*	0.03*	0.15

Table 6 presents antibiotic prescriptions by physicians according to common presenting symptoms of URTI. Antibiotic prescription was used by 63 (45%) of the studied physicians for patients presented with running noses. About two-thirds of physicians were prescribing antibiotics for patients presented with fever and sore throat and tympanic membrane redness. A total of 118 (84.2%) physicians prescribed antibiotics for patients with ear discharge, congested tonsils, and tender upper cervical lymph nodes.

**Table 6:** Antibiotic prescription by physicians according to common presenting symptoms of upper respiratory tract infection (n= 140) Data has been presented as frequencies(n) and percentages (%).

Symptoms/signs*	N (%)
Running nose	63 (45.0)
Fever	95 (67.9)
Sore throat	93 (66.4)
Cough	55 (39.31)
Ear pain	35 (25.0)
Ear discharge	118 (84.2)
Congested tonsils	122 (87.1)
Tympanic membrane redness	92 (65.7)
Tender upper cervical lymph nodes	125 (89.3)

Table 7 presents the frequency distribution of patients' requests for antibiotics for URTI. It revealed that 60 (42.8%) were asked to prescribe antibiotics 1-3 times over one month. About 38 (27.1%) of the studied physicians were asked to prescribe antibiotics 4-7 times and 22 (15.7%) of them were asked to prescribe antibiotics more than seven times. Only 20 (14.4%) were not asked to prescribe antibiotics to the patients with URTIs.

**Table 7:** Frequency of patients' requests for antibiotics for URTI Data has been presented as frequencies (n) and percentages (%). URTI: upper respiratory tract infection

Request*	N (%)
Never	20 (14.4)
1-3	60 (42.8)
4-7	38 (27.1)
> 7	22 (15.7)

Table 8 presents the response of physicians to patient's requests and advice to prescribe antibiotics. A total of 89 (63.6%) rarely prescribed antibiotics when a patient requests and about 28 (20%) of them sometimes prescribed antibiotics when a patient requests. The number of physicians who advise their patients on simple self-management was 96 (68.6%) and about 55 (39.3%) always advise patients on simple medication and antibiotics.

**Table 8:** Response of physicians to patient's requests and advice (n=140) Data has been presented as frequencies (n) and percentages (%).

Physicians' response*	Always n (%)	Sometimes n (%)	Rarely n (%)	Never n (%)
The physician prescribes antibiotics when requested by a patient.	3 (2.1)	28 (20.0)	20 (14.3)	89 (63.6)
The physician advises the patient on simple medications.	96 (68.6)	30 (21.4)	8 (5.7)	6 (4.3)
The physician advises the patient on simple medications and antibiotics.	55 (39.3)	50 (35.7)	17 (12.1)	18 (12.9)

Table 9 displays the factors affecting the decision of the studied physicians to prescribe antibiotics for patients with URTIs by their title. The studied factors showed no statistically significant differences between the studied physicians by their title (general practitioners vs. specialists (p= 0.65)). The main non-clinical factor that affected the general practitioners' decision to prescribe antibiotics was the patient's request, 51 (54.8%). The specialists as well were highly influenced by patients' requests, 23 (53.6%).

**Table 9:** Non-clinical factors affecting the decision of the studied physicians to prescribe antibiotics for patients with URTIs by their title (n=140). Data has been presented as frequencies(n) and percentages (%). * P-value considered statistically significant at p-value< 0.05. URTIs: upper respiratory tract infections

Factors*	General practitioners (n= 93), N, %	Specialists (n= 47), N, %	P-value
Patients' request	51 (54.8)	23 (53.6)	0.65
Patients' press	19 (20.4)	9 (21.8)	
Work pressure	10 (10.7)	4 (9.3)	
Not seeing the patients again	5 (5.4)	2 (4.7)	
Expecting patients' re-consultation if not get better and/or are not prescribed antibiotics	8 (8.7)	5 (11.6)	

## Discussion

​​​​While antibiotics are deemed suitable for addressing acute bacterial URTIs, their effectiveness in treating viral URTIs has been proven to be inadequate [16]. Several earlier studies have proposed that the majority of URTIs are self-limiting, necessitating symptomatic treatment, and that the administration of antibiotics is more likely to pose harm to patients rather than provide benefit [17]. The current study has studied the behavior of PHC physicians toward antibiotic prescriptions for URTIs in Al-Madinah city, Saudi Arabia.

The study findings revealed that the majority, 62 (44.3%) of physicians prescribed antibiotics to almost half of their URTI patients. A similar study conducted in Malaysia by Teng et al. found that antibiotics were prescribed mostly in patients with URTIs (68.4%) than those without URTIs [18]. In Wisconsin and Minnesota, USA study, 60% of the adults and 46% of the children had antibiotics prescriptions for non-bacterial infections [19]. These results indicate a high prevalence of antibiotic course prescriptions for URTIs among PHC physicians which is alarming and necessitates focusing and understanding of the problem to be able to develop effective intervention programs to decrease this phenomenon.

This study further sought to determine the distinct antibiotic prescription patterns based on professional designations. Among the general practitioners, amoxicillin stood out as the most prescribed antibiotic, by 60 (64.5%) of the practitioners, followed by Augmentin, 20 (21.5%). On the other hand, specialists exhibited a different trend with 22 (46.8%) prescribing amoxicillin and 20 (42.6%) prescribing Augmentin. Erythromycin showed minimal usage across both groups. Quinolones and cephalosporins displayed marginal differences, with 5.4% and 6.3% utilization among general practitioners and specialists, respectively. Statistical analysis demonstrated significant discrepancies in antibiotic prescription patterns between the two groups for amoxicillin (p=0.01*) and Augmentin (p=0.04*), emphasizing distinctive preferences in antibiotic choices based on professional roles among PHC physicians. These results align with a similar study conducted in Ghana that revealed the profession of the practitioner was a predictor of the type of antibiotic that is prescribed to patients [20].

Most physicians claimed that ear discharge, congested tonsils, and tender upper cervical lymph nodes correlate significantly with higher antibiotic prescriptions, suggesting a strong inclination among physicians to prescribe antibiotics when these symptoms are present. Conversely, symptoms such as running nose, cough, and ear pain show notably lower antibiotic prescription rates, indicating a more cautious approach to antibiotic use for these manifestations, potentially aligning with guidelines promoting judicious antibiotic utilization in cases where symptoms may not necessarily warrant antibiotic treatment. Similar findings were observed in a Saudi study where the strongest indication for antibiotic prescription was the presence of throat exudates, with 86% of physicians opting to prescribe antibiotics for patients with cervical lymphadenopathy [21].

Regarding patients' requests for antibiotics, various studies explored this aspect. Some authors attributed the inclination to fulfill such requests to younger physicians who might aim to establish their practice by meeting patient demands for antibiotics [22]. In this current study, a total of 89 (63.6%) physicians seldom prescribed antibiotics upon patient requests, while around one-fifth of them occasionally did so.

This study further found the majority of physicians, 96 (63.6%) rarely or never prescribe antibiotics when directly requested by patients, suggesting a tendency to prioritize clinical judgment over patient demands. There was no statistically significant relationship between patients' requests and pressure to prescribe antibiotics, however, factors such as work pressure, not expecting to see the patients again, and anticipating re-consultation if antibiotics aren't prescribed exhibited minimal discrepancies between the two groups, suggesting that these non-clinical influencers impact physicians' antibiotic prescription behaviors irrespective of their professional titles within the workplace. This result is in contrast to what was reported in a rural China study, where 70% of the village doctors complied with the primary caregivers’ request and pressure even when they felt the antibiotics were unnecessary [23]. This difference could be due to the differences in cultures and beliefs among communities.

Nevertheless, this study was marred by some limitations. Self-selection bias may have been a limitation factor in this study because those subjects who chose to participate may be more familiar with WHO guidelines for antibiotics prescription for patients with URTIS than those who refused to be contacted. Also, this study did not include some important factors related to the attending patients that may affect the physician’s decision to prescribe antibiotics to them. However, because the scope and objective of this study were restricted to studying the behavior among the physicians; these factors could be studied in separate studies in the future.

## Conclusions

The finding of the current study shed light on the problem of inappropriate antibiotic prescription in primary care centers, where the majority of physicians prescribed antibiotics to their patients, where the main non-clinical reason to prescribe antibiotics was patients’ request. The most commonly prescribed antibiotics were amoxicillin followed by Augmentin, which is in contrast to the guidelines. The main duration of using antibiotics was lower than in other international studies. Congested tonsils, ear discharge, and cervical lymphadenopathy were the most common clinical factors that affected physicians' decisions to prescribe antibiotics for URTI. There was no significant difference between general physicians and specialists regarding the influence of non-clinical factors on their decision to prescribe antibiotics.
